# Rules of Expansion: an Updated Consensus Operator Site for the CopR-CopY Family of Bacterial Copper Exporter System Repressors

**DOI:** 10.1128/mSphere.00411-20

**Published:** 2020-05-27

**Authors:** Henrik O’Brien, Joseph W. Alvin, Sanjay V. Menghani, Yamil Sanchez-Rosario, Koenraad Van Doorslaer, Michael D. L. Johnson

**Affiliations:** a Department of Immunobiology, University of Arizona, Tucson, Arizona, USA; b BIO5 Institute, University of Arizona, Tucson, Arizona, USA; c School of Animal and Comparative Biomedical Sciences, Cancer Biology Graduate Interdisciplinary Program, Genetics Graduate Interdisciplinary Program, University of Arizona Cancer Center, University of Arizona, Tucson, Arizona, USA; d Valley Fever Center for Excellence, University of Arizona, Tucson, Arizona, USA; University of Iowa

**Keywords:** copper, metal, operator, operon, protein, repressor

## Abstract

Copper is broadly toxic to bacteria. As such, bacteria have evolved specialized copper export systems (*cop* operons) often consisting of a DNA-binding/copper-responsive regulator (which can be a repressor or activator), a copper chaperone, and a copper exporter. For those bacteria using DNA-binding copper repressors, few studies have examined the regulation of this operon regarding the operator DNA sequence needed for repressor binding. In Streptococcus pneumoniae (the pneumococcus), CopY is the copper repressor for the *cop* operon. Previously, homologs of pneumococcal CopY have been characterized to bind a 10-base consensus sequence T/GACANNTGTA known as the *cop* box. Using this motif, we sought to determine whether genes outside the *cop* operon are also regulated by the CopY repressor, which was previously shown in Lactococcus lactis. We found that S. pneumoniae CopY did not bind to *cop* operators upstream of these candidate genes *in vitro*. During this process, we found that the *cop* box sequence is necessary but not sufficient for CopY binding. Here, we propose an updated operator sequence for the S. pneumoniae *cop* operon to be ATTGACAAATGTAGAT binding CopY with a dissociation constant (*K_d_*) of ∼28 nM. We demonstrate strong cross-species interaction between some CopY proteins and CopY operators, suggesting strong evolutionary conservation. Taken together with our binding studies and bioinformatics data, we propose the consensus operator RNYKACANNYGTMRNY for the bacterial CopR-CopY copper repressor homologs.

**IMPORTANCE** Many Gram-positive bacteria respond to copper stress by upregulating a copper export system controlled by a copper-sensitive repressor, CopR-CopY. The previous operator sequence for this family of proteins had been identified as TACANNTGTA. Here, using several recombinant proteins and mutations in various DNA fragments, we define those 10 bases as necessary but not sufficient for binding and in doing so, refine the *cop* operon operator to the 16-base sequence RNYKACANNTGTMRNY. Due to the sheer number of repressors that have been said to bind to the original 10 bases, including many antibiotic resistance repressors such as BlaI and MecI, we feel that this study highlights the need to reexamine many of these sites of the past and use added stringency for verifying operators in the future.

## Abstract (precis)

Many Gram-positive bacteria respond to copper stress by upregulating a copper export system controlled by a copper-sensitive repressor, CopR-CopY. The previous operator sequence for this family of proteins had been identified as TACANNTGTA. Here, using several recombinant proteins and mutations in various DNA fragments, we define those 10 bases as necessary but not sufficient for binding and in doing so, refine the *cop* operon operator to the 16-base sequence RNYKACANNTGTMRNY. Due to the sheer number of repressors that have been said to bind to the original 10 bases, including many antibiotic resistance repressors such as BlaI and MecI, we feel that this study highlights the need to reexamine many of these sites of the past and use added stringency for verifying operators in the future.

## INTRODUCTION

Metals are essential nutrients to all living organisms. Roughly 40% of proteins use metal as cofactors and structural components in a vast number of cellular processes (1). Iron, zinc, and manganese are examples of first-row divalent transition metals used by living organisms. The ability to form stable complexes plays a vital role as to how each metal is used in the organism. The stability of protein-metal complexes is generalized by the Irving-Williams series (Mn < Fe < Co < Ni < Cu > Zn) (2). In general, more stable complexes correlate to a metal’s toxicity, as native metals for an active site can be displaced by another metal ion further along in the observed series. This mismetallation is present across multiple metal-binding motifs and can result in abnormal protein function (3). For most prokaryotes, however, metals such as copper, nickel, and cobalt are broadly toxic. Higher order organisms have evolved ways to regulate and transport these metals, reducing promiscuity in mature active sites. Mammalian hosts have evolved strategies to both sequester essential metals from bacteria (e.g., Fe, Mn, and Ca) and bombard them with toxic metals such as copper; a strategy called nutritional immunity (4, 5). Mutations or deletions of a mammalian copper transporter ATP7A can attenuate bacterial clearance by macrophages (6, 7). Thus, both within mammalian systems and on surfaces, copper is utilized as an antimicrobial (8–12).

Copper can catalyze Fenton-like reactive oxygen stress in bacteria, but mismetallation of iron-sulfur clusters and enzymes necessary for nucleotide and amino acid synthesis also leads to significant toxicity (13–17). As such, bacteria have evolved specialized import and export systems to acquire necessary metals and to adapt to this metal toxicity. The presence of these import systems within the bacteria typically indicates a nutritional requirement for the metal. Iron, for instance, is an essential metal for Streptococcus pneumoniae (the pneumococcus), a Gram-positive pathogen that causes pneumonia, meningitis, otitis media, and septicemia. In S. pneumoniae, iron has five known import systems/contributing genes, Pia, Piu, Pit, the hemin-binding system encoded by *SPD_1590* (D39 strain), and *SPD_1609*, but no known export systems (18–20). Calcium, zinc, and manganese all have export and import systems, which suggests that there are many optima of concentrations depending on the cell requirements. However, the pneumococcus has no known import system for copper but contains a dedicated copper export system encoded by the *cop* operon (19, 21–24). In general, copper export systems consist of an operon DNA regulator, a copper chaperone, and one or two copper exporters (21, 25–31).

Multiple studies regarding the *cop* operon have been performed in S. pneumoniae (14, 21, 30, 32–35) and in other bacteria. Globally, *cop* operon regulators function as either repressors, as in S. pneumoniae, or activators. Although there are *cop* operon activators and repressors in structurally distinct groups, they all serve to protect the bacteria against copper stress by sensing copper and facilitating its export. Activators, such as CueR in Escherichia coli, sense copper and bind upstream of the *cop* operon to promote transcription (36). Conversely, repressor proteins release DNA upon binding copper and are found in species such as Lactococcus lactis and S. pneumoniae (CopR-CopY), Listeria monocytogenes, and Mycobacterium tuberculosis (CsoR) (19, 27, 30, 34, 35, 37). The pneumococcal *cop* operon contains, *copY* as the DNA repressor, *cupA* as a membrane-associated copper chaperone, and *copA* as the copper-specific exporter (19, 30, 34). The CopY repressor protein has an N-terminal helix-wing-helix motif, which directs the homodimer to bind its cognate DNA (34, 35). The Cu-chaperone protein CupA chelates intracellular Cu, reduces it from Cu^2+^ to Cu^1+^, and finally delivers it to CopA for export (32–34, 38). CupA copper chelation allows for the recycling of CopY to the operator when copper is exhausted. Mutations in the copper export protein in *cop* operons result in decreased bacterial virulence, highlighting the importance of copper in nutritional immunity (21, 30, 39, 40).

Pneumococcal CopY is homologous to several known antibiotic resistance repressors, including BlaI, a Staphylococcus aureus MecI homolog that represses the gene for a β-lactamase (34, 35, 41). Like CopY, BlaI and MecI interact with a known operator sequence, TACA/TGTA, form homodimers, and are mostly helical in secondary structure (34, 41). However, BlaI is regulated by proteases instead of metal interactions (42).

The CopR-CopY family of *cop* operon repressors have C-terminal metal-binding protein motifs, Cys-X-Cys. Each CXC motif binds copper in a 1:1 ratio. These motifs can also bind zinc with a stoichiometry of two CXC motifs to one zinc (35). Since these proteins function as dimers, the stoichiometry is one zinc per dimer, or two coppers per dimer after zinc displacement. Copper binding to CopR-CopY proteins causes a conformational change, leading to release from the operator site, while zinc binding leads to higher *cop* operator affinity (34, 35). However, how binding metal directly leads to the conformational changes associated with DNA binding is currently unknown. Previous work in Enterococcus hirae demonstrated that the CopY protein binds to two palindromic regions (TACANNTGTA) upstream of the *cop* operon by using a DNase-footprinting assay (25). The *cop* operator sequence was then identified using surface plasmon resonance to evaluate binding of CopR-CopY to promoter regions in *E. hirae*, Lactococcus lactis, and Streptococcus mutans that contained TACANNTGTA (43). The oligonucleotides used in this study contained a promoter sequence beyond just the 10 bases listed above. We suspect that the 10 bases were identified as the operator sequence based on the palindromic nature of the sequence, without testing whether those bases alone were sufficient for CopY binding.

Since the CopR protein from L. lactis was found to regulate multiple genes, we sought to test potential CopY-regulated genes outside the *cop* operon in Streptococcus pneumoniae (44). However, we quickly found that the TACANNTGTA operator is not a reliable predictor for repressor protein binding (44). We used S. pneumoniae CopY in binding experiments with various DNA constructs to define a sufficient *cop* operator *in vitro*. Here, we present our findings and propose an update to the *cop* operator motif. We found that unlike the LacI repressor system, pneumococcal CopY does not demonstrate any cooperative binding, despite the operator sites being in close proximity. Furthermore, we highlight the strong conservation between bacterial *cop* operators and CopR-CopY constructs by demonstrating the presence and strength of cross-species interactions. We identified bacteria with CopY homologs and aligned the upstream sequences to determine if they also contained putative *cop* operators and whether these operators followed the consensus motif we have proposed.

## RESULTS

### CopY operator homology.

**FIG 1:**
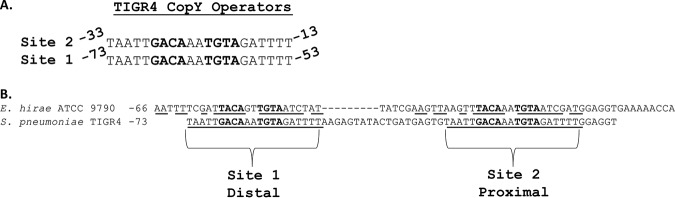
TIGR4 has two 21-base repeats containing the consensus CopY operators. (A) Aligned 21-base sequences for the two CopY operators. (B) TIGR4 *SP_0727* promoter region sequence (containing both 21-base repeats) aligned with the *E. hirae* ATCC strain 9790. Identical bases are underlined for the respective regions containing the operator.

Early DNA-binding studies were carried out using a CopY homolog from Enterococcus hirae on the interactions with the *cop* operon operator (25, 43, 45). We observed that there are two large repeats upstream of the pneumococcal *cop* operon that include a 10-base sequence important for CopY binding. These motifs in S. pneumoniae differed slightly from those observed in *E. hirae* (Fig. 1A). Although the amino acid sequence of the *E. hirae* copper repressor and the upstream binding repeats are highly similar to those of S. pneumoniae (34, 35) and contain the 10-base sequence, *E. hirae* operators upon initial observation lacked the extended regions flanking this sequence in pneumococcus (Fig. 1B) (25). A BLAST search revealed that the 61-base stretch of DNA upstream of the pneumococcal *cop* operon that includes the two extended 21-base repeats is highly conserved in all pneumococcal strains (46).

### CopY binds to both pneumococcal *cop* operators.

**FIG 2:**
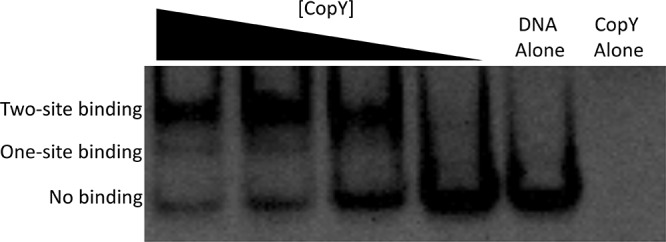
CopY binds to both *cop* operon operators. EMSA with CopY and wild-type DNA. In seven of eight wells, a final concentration of 50 nM DNA was used with protein concentrations titrated by 2.5-fold dilutions (640, 256, 102, and 41 nM). A final concentration of 640 nM CopY was used in a protein-without-DNA control with each replicate.

Previous studies showed that CopR-CopY specifically bound to the *cop* operon operator in sequence- and metal-dependent manners, as disrupting the operator bases or adding copper disrupted binding, while adding manganese or iron had no detectable effect (34). These studies were conducted with only one full T/GACANNTGTA motif (here, KACANNTGTA) intact (34, 35). It is not clear why these operators are duplicated or why they fail to adhere to strict palindrome sequences. We preliminarily sought to first determine if CopY bound to a DNA fragment containing both operators and if we could observe binding to one or both operators. Using a 61-base double-stranded DNA (dsDNA) fragment and recombinant CopY, we used an electrophoretic mobility shift assay (EMSA) to measure binding. As expected, CopY binds in a dose-dependent manner to the 61-base dsDNA fragment (Fig. 2). Furthermore, there were two shifts displayed on the EMSA, indicating that the second-site interaction was concentration dependent (Fig. 2). These data are in agreement with initial studies of CopR in *E. hirae*, which also contains two operator sites.

**FIG 3:**
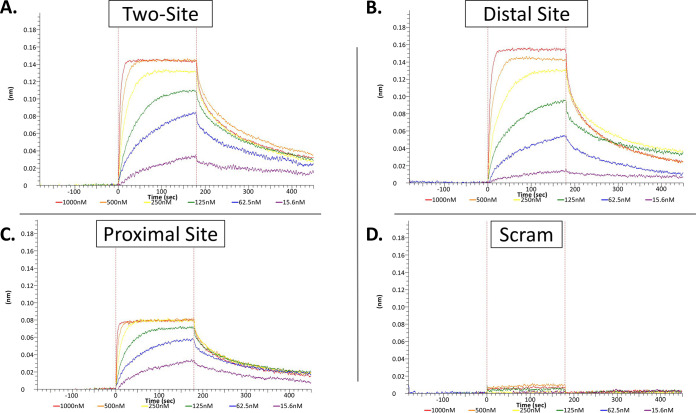
Affinity measurements for CopY and the *cop* operon operators. DNA fragments were loaded onto a biosensor and tested with 1,000 nM (red), 500 nM (orange), 250 nM (yellow), 125 nM (green), 62.5 nM (blue), and 15.6 nM (purple) CopY wild type (A), distal site (B), proximal site (C), or scrambled (scram) (D). For each panel, data are representative of at least three experimental replicates.

To quantitatively determine the affinities to the operators, we used a higher throughput system to determine affinity: a biolayer interferometry (BLI) assay using the Octet Red384 (Octet). BLI is an optical technique similar to surface plasmon resonance that uses interference of white light reflected from two surfaces to measure interactions between molecules but is performed in a 96- or 384-well plate (47). The process involves (i) a baseline step in buffer; (ii) a step for the loading of analyte A to the surface of a biosensor; (iii) a second baseline step to make sure analyte A stays on the sensor (which should appear flat); (iv) an association step of analyte B (which should appear as a shift in reflected light if binding occurs); and (v) a disassociation step (which should appear as an opposite shift in the reflected light as analyte A disassociates from analyte B). In our experiments, analyte A was the biotinylated dsDNA oligonucleotides which bind to the streptavidin biosensor, and analyte B was the recombinant protein (CopY). For viewing simplicity, we begin our plots begin at step 3. These experiments demonstrated that the two-operator-site (here, wild type) DNA had a similar affinity (dissociation constant [*K_d_*] = 28.1 nM) as that of the proximal DNA (DNA that has the distal site scrambled) (*K_d_* = 25.5 nM) to CopY (Table 1; Fig. 3A and C). The distal site DNA (DNA that has the proximal site scrambled) had a slightly lower affinity (*K_d_* = 55.2 nM) (Table 1; Fig. 3B). Since the sequences are identical, we suspect this difference is because the first 5′ base in that motif is linked to biotin, in turn bound to the streptavidin-coated biosensor. Thus, we would encourage including more than one base between the DNA binding sequence and protein. As expected, CopY bound DNA constructs containing intact 21-base repeat-containing *cop* operon operators with significantly higher affinities than for the scrambled DNA sequence (scram) (Fig. 3D) or single-stranded DNA (ssDNA) containing the wild-type operators, which both exhibited extremely weak binding (data not shown). Taken together, CopY binds both 21-base repeats containing the operator sites independently of each other with nanomolar affinity.

**TABLE 1:** Data and model statistics from Octet kinetic experiments

Construct	*K_d_* (nM)^*a*^	*R*^2^
Wild type	28.1 ± 0.2	0.9474
Distal site	55.2 ± 0.4	0.9432
Proximal site	25.5 ± 0.2	0.9475
16 bp	360.0 ± 3.0	0.9808
19 bp	37.1 ± 0.3	0.9552
19 bp T to C	40.4 ± 0.3	0.9408
Full palindrome	39.6 ± 0.4	0.9056
*E. hirae* proximal	164.0 ± 2.0	0.9269

a The *K_d_* and model fit of dsDNA fragments containing wild-type or variations of the *cop* operator to recombinant S. pneumoniae CopY were assessed using BLI. At least 3 replicates were performed for each construct.

### Defining the pneumococcal CopY operator.

**FIG 4:**
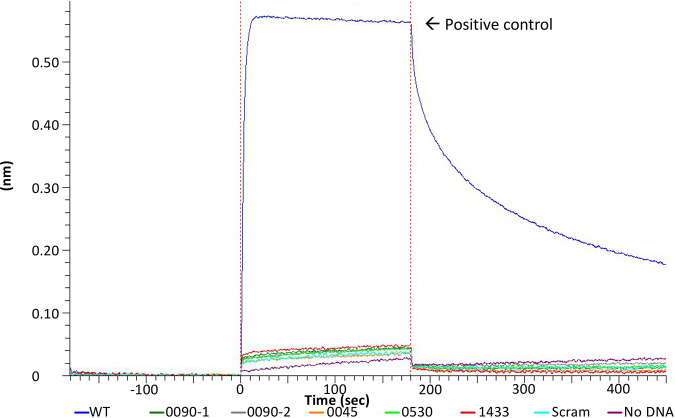
Prediction of CopY binding based on 10-base sequence overestimates binding sites. CopY at 3 μM was used to assess binding to DNA fragments containing potential CopY operators upstream of the respective genes *SP_0090* 1 (green), *SP_0090* 2 (gray), *SP_0045* (orange), *SP_0530* (light green), *SP_1433* (red), with controls for wild type (blue), scrambled (scram; light blue), and no DNA (purple). Data are representative of three experimental replicates.

**FIG 5:**
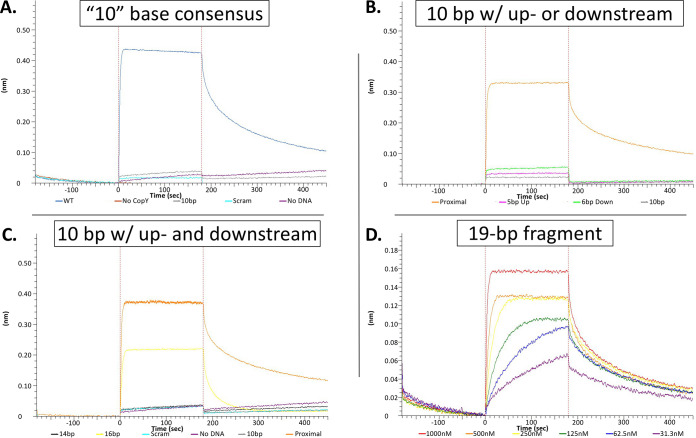
The minimum operator for sufficient CopY binding is 16 bases in length. (A) Binding of the 10-base fragment compared to positive and negative controls: wild type (blue), Wild type no protein (red), 10-base sequence (gray), scrambled (scram; light blue), and no DNA (purple). (B) DNA fragments containing either the upstream (5 bases) or downstream (6 bases) of the 10-base sequence within 21-base repeat compared to positive and negative controls: proximal site (orange), five bases upstream (pink), six bases downstream (light green), 10-base sequence (gray). (C) Fragments were used to assess extended sequence on both sides of the 10-base sequence: proximal site (orange), 14 bases (black), 16 bases (yellow), 10 bases (gray), scram (light blue), and no DNA (purple). For data in panels A to C, we used 3 μM CopY to assess binding to each of the fragments. (D) A fragment containing 19 of the 21 bases in the repeat had comparable levels of binding to the full repeat. The following concentrations were used to establish the *K_d_*: 1,000 nM (red), 500 nM (orange), 250 nM (yellow), 125 nM (green), 62.5 nM (blue), and 31.3 nM (purple) CopY. Data are representative of three experimental replicates.

With a reported binding sequence of KACANNTGTA, we hypothesized that CopY may also bind similar sequences, as previously seen in Lactococcus lactis (44). Allowing for one base variation from the reported binding sequence, we found matches upstream of genes upregulated under copper stress and hypothesized that they may also be regulated by CopY (14, 48). Seven potential binding sites were assessed using BLI. To our surprise, CopY did not bind to these proposed operator sites (Fig. 4 and Table 2; see also Fig. S1 in the supplemental material). Based on these results, we suspected that the reported sequence in the literature may be necessary, but not sufficient, for CopY binding. Indeed, we found that CopY failed to bind the reported binding sequence (Fig. 5A). Taken together, we have concluded that the reported binding operator is necessary but not sufficient for *cop* repressor binding (25).

**TABLE 2:** Outcomes of CopR or CopY binding to potential operator sites from L. lactis (44) or S. pneumoniae, respectively

Organism	Color in Fig. 4	Closest downstream gene	Sequence^*a*^	CopY-CopR binding	Reference
Lactococcus lactis	NA^*b*^	*ytjD1*	AAATAGTT**TACA**AG**TGTA**AATTTATTT	Yes	44
	NA	*ydiD*	AAAATGTT**TACA**TG**TGTA**AATTTTCAC	Yes	44
	NA	*copR*	TTAGTGTT**TACA**CG**TGTA**AACTTATCT	Yes	44
	NA	*copB*	TGATAGTT**TACA**AT**TGTA**AACTATATA	Yes	44
	NA	*yahC*	TTTTCGTT**TACA**AT**TGTA**AACATAGAA	Yes	44
	NA	*lctO*	CTATCATC**TACA**GA**TGTA**AACTTTATA	Yes	44
	NA	*ytjD2*	GATAAGAT**TACA**TA**TGTA**AACAATAAA	Yes	44
	NA	*yfhF*	TAAGTATA**TACA**TC**TGTA**AAACTGAAA	No	44
	NA	*yxdE*	TTTGCTAT**TACA**CT**TGTA**TCACATAAA	No	44
Streptococcus pneumoniae	Dark green	*Sp_0090 1*	TGATTTAG**GACA**TT**TGTT**TGATAGTGG	No	This study
	Gray	*Sp_0090 2*	GAGTATAC**TAAT**AA**TGTA**ATCGTTATC	No	This study
	Orange	*Sp_0045*	GGTGAACT**AACA**GA**TGTT**TACGAAATT	No	This study
	Light green	*Sp_0530*	ATTTGAGG**AACA**AA**TGTA**CGTTTATAA	No	This study
	Red	*Sp_1433*	GTAATTAT**AACA**GA**TGTA**TAATAGAAA	No	This study
	NA	*Sp_1863*	ATGAATAA**AACA**AT**TGTA**ACACTCATC	No	This study
	NA	*Sp_2073*	AAGGCGGA**AACA**TG**TGTC**AATGACTTG	No	This study
	NA	CopY (proximal site)	GTGTAATT**GACA**AA**TGTA**GATTTTGGA	Yes	This study
	NA	CopY (distal site)	CTATAATT**GACA**AA**TGTA**GATTTTAAG	Yes	This study

a Underlined bases indicate bases varying from the reported 10-base consensus sequence (bold font).

b NA, not applicable.

10.1128/mSphere.00411-20.1 FIG S1 CopY does not bind predicted sites continued. CopY at 3 μM was used to assess binding to DNA fragments containing potential CopY operators upstream of the respective genes *SP_1863* (gray) and *SP_2073* (orange), with controls, WT DNA (blue, positive control) and scram (light blue, negative control). Download FIG S1, PDF file, 0.2 MB. Copyright © 2020 O’Brien et al. 2020 O’Brien et al. This content is distributed under the terms of the Creative Commons Attribution 4.0 International license.

We next wanted to establish which bases outside the previously reported 10-base KACANNTGTA sequence were necessary for CopY binding. Beginning with the minimal 10-base sequence, constructs for BLI were designed to include or exclude identical bases from the 21-base S. pneumoniae CopY operator. The first set of sequences extended the operator five bases upstream or six bases downstream of the included 10-base sequence. The remaining positions were left scrambled. Neither of these fragments bound S. pneumoniae CopY more than the negative controls, suggesting there are essential bases on each side of the 10-base sequence (Fig. 5B). We then widened the operator construct by two or three bases on either side of the core 10-bases for a total of 14 and 16 bases of the total 21 bases. These operators were used to determine the minimum operator size for S. pneumoniae CopY. The 14-base sequence did not display binding greater than that of negative controls (*K_d_* > 3 μM), but the 16-base sequence displayed approximately ∼10-fold weaker affinity than the proposed “full-length” 21-base operator. (Fig. 5C and Table 1; see Fig. S2). Extending the sequence to 19 of the 21 bases led to comparable levels of binding to the full sequence (Fig. 5D). Based on these data, we suggest that 16 bases make up a minimal operator site (ATTGACAAATGTAGAT) recognized by CopY and that additional flanking bases increase stability.

10.1128/mSphere.00411-20.2 FIG S2 Distances in bases between operators in species that had two operators. A python script was generated to count and plot on a graph the number of bases between operators (available at https://github.com/Van-Doorslaer/Alvin_et_al_2018). Download FIG S2, PDF file, 0.1 MB. Copyright © 2020 O’Brien et al. 2020 O’Brien et al. This content is distributed under the terms of the Creative Commons Attribution 4.0 International license.

### Bioinformatic characterization of the CopY-CopR operator.

We performed BLASTp searches for S. pneumoniae TIGR4 CopY homologs, first excluding and then including the *Streptococcus* genus (46). Using a maximum target sequence number of 1,000 for each search, and then combining both lists, we found 335 candidate homologs (see Data Set S1, Tab 1). From this list, we extracted protein sequences from the unique NCBI accession numbers—many NCBI accession numbers represented several and/or identical homologs. Many of the 141 unique protein sequences belonged to species in the *Streptococcus* and *Lactobacillus* genera (Data Set S1, Tab 2). This table also included species such as the yogurt probiotic Lactobacillus acidophilus and Mycobacteroides abscessus, an emerging multidrug-resistant pathogen that causes lung, skin, and soft tissue infections (49).

10.1128/mSphere.00411-20.5 DATA SET S1 (Tab 1) Homologous protein sequences to S. pneumoniae TIGR4 CopY from BLAST search. (Tab 2) Homologous protein and DNA sequences to S. pneumoniae TIGR4 CopY with unique protein accession numbers. (Tab 3) Homologous protein and DNA sequences to S. pneumoniae TIGR4 CopY with unique genome accession numbers. (Tab 4) CopY consensus *cop* operon operators from MEME suite from unique genome accession numbers. (Tab 5) CopY consensus *cop* operon operators from Tab 4 with only one *cop* operon operator. (Tab 6) Organisms from Tab 4 with no consensus *cop* operon operator. Download Data Set S1, XLSX file, 0.1 MB. Copyright © 2020 O’Brien et al. 2020 O’Brien et al. This content is distributed under the terms of the Creative Commons Attribution 4.0 International license.

**FIG 6:**
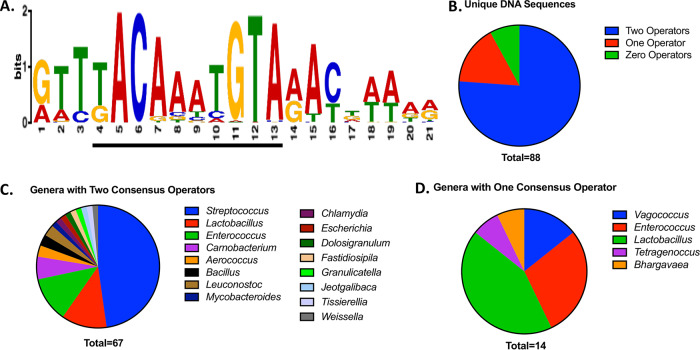
CopY *cop* operon operator consensus sequences in the genomic DNA. (A) Top consensus DNA sequence contained within the 87 unique upstream 100 bases sequences to the respective CopY organism as detected by MEME suite. The literature-based T/GACANNTGTA is underlined within the program-generated consensus sequence. (B) Total unique DNA sequences from Data Set S1, Tab 4, listed by number of CopY operators. (C) Unique genera with two CopY *cop* operon operators from Data Set S1, Tab 4. (D) Unique genera with one CopY operator from Data Set S1, Tab 5.

We used programs within the MEME suite to identify DNA motifs within 100-bases upstream of the *copY* homolog start codon, which would correspond to the promoter and suspected operator-containing elements (Data Set S1, Tab 3) (50, 51). Similar to that of known CopR-CopY operators, the program-derived 21-base sequence contained KACANNTGTA (Fig. 6A) (52). Of these 88 sequences, 67 had two CopR-CopY *cop* operon operators (similar to those in Streptococcus pyogenes, *E. hirae*, and Enterococcus faecium), 14 had one operator (such as Enterococcus faecalis), and 7 lacked detectable sequence similarity (Fig. 6B to D; Data Set S1, Tab 4 to 6). Increasing the maximum bases upstream to 500 did not yield additional sequences (data not shown). For the genomes with two operators, we found that most of the operators were between 24 and 39 bases from each other, with the mode being 26 bases (Fig. S2). Of the species that had a single *cop* operon operator, the sequences were more variable KACANNYGTA (Data Set S1, Tab 5). Of these 14 sequences, 7 were on the positive strand (but six were palindromic), and 7 nonpalindromic sequences were on the negative strand (Data Set S1, Tab 5). Of the seven sequences that had no apparent operator homology, six were in the *Lactobacillus* genus and one was in the *Macrococcus* genus (Data Set S1, Tab 6). Comparing the upstream regions across these seven strains, MEME suite did not identify a consensus motif. This suggests that these seven strains lost the operator since diverging from a common ancestor.

### Determining the consensus *cop* operon operator.

**FIG 7:**
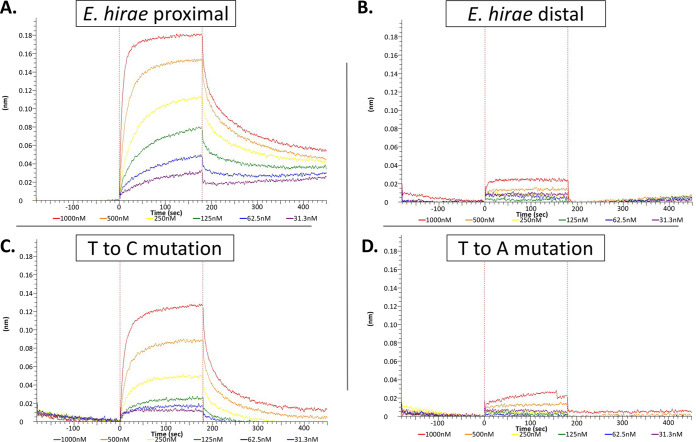
Pneumococcal CopY binds to *E. hirae* DNA in accordance with the newly proposed consensus *cop* operon operator. Affinity of CopY binding to various DNA fragments was determined using the following concentrations of CopY: 1,000 nM (red), 500 nM (orange), 250 nM (yellow), 125 nM (green), 62.5 nM (blue), and 31.3 nM (purple). (A) *E. hirae* proximal site. (B). *E. hirae* distal site (C) The 19-base fragment with a T-to-C mutation. (D) The 19-base fragment with a T-to-A mutation. For each panel, data are representative of three experimental replicates.

The initial characterization of the CopY operator sites was performed using a few mutations in the *E. hirae cop* promoter (25, 53). As a proof of principle, we used the native *E. hirae cop* distal or proximal operators and examined them for pneumococcal CopY binding (Fig. 7A and B). S. pneumoniae CopY bound to the proximal *E. hirae* operator with low nanomolar affinity (Fig. 7A). However, pneumococcal CopY did not bind to the distal *E. hirae* operator (Fig. 7B). This implies that for CopY from S. pneumoniae, the central “AA” motif (previously notated as “NN”) cannot accommodate variations at this position (Fig. 7B).

While inspecting the alignment of bacterial species operator sites via MEME Suite (Fig. 1A and 6A; Data Set S1, Tab 4), we identified a clear purine-N-pyrimidine pattern on each side of the previous 10-base operator. Using our sequence data of homologous *cop* operators, we arrived at a proposed consensus sequence of RNYKACANNTGTARNY (where “R” is purine, “Y” is pyrimidine, and “K” is either G or T) (44) for CopY family repressor operators. These data were supported by both the *E. hirae cop* operators having the alternate purine or pyrimidine bases at positions 1, 14, and 16 but are still functional (Fig. 1B). Thus, we focused on position 3, which was the same in the pneumococcal operator and *E. hirae* operators in order to test our hypothesis that conserved R-N-Y motifs flank the 10-base core at positions 1, 3, 14, and 16. Position 3 was mutated to the alternate pyrimidine (T to C) or switched from pyrimidine to purine (T to A). As expected from our model, the T to C mutation at position 3 did not significantly alter the binding affinity compared to that of the 19-base fragment, while the T to A mutation completely abolished binding (Fig. 7C and D).

**FIG 8:**
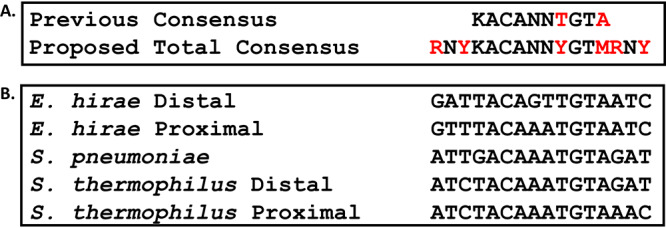
Chart representing the previous, newly proposed pneumococcal, and newly proposed CopR-CopY protein family consensus *cop* operon operator. (A) Bases that change from the initial 10-base consensus operator in the now total consensus operator are highlighted in red. (B) Sequence alignment of the S. pneumoniae, S. thermophilus, and *E. hirae* operator sites.

Due to the palindromic nature of this operator, we decided to test the last nonpalindromic base in the now RNYKACAAATGTARNY consensus which was the “A” in position 13. With position 4 being K (G or T), we mutated the “A” at position 13 to a “C” so that would fit with the complementarity compared to position 4, termed “full palindrome.” We found that pneumococcal CopY was able to bind to this full palindrome sequence at near WT levels (Table 1). Taken together, we believe that the pneumococcal DNA binding sequence for CopY is RNYKACAAATGTMRNY, with “M” representing “C” or “A,” as opposed to the previously reported KACANNTGTA (Fig. 8A).

### Interspecies compatibility *in vitro* across CopY family proteins and operators.

Thus far, we have defined the pneumococcal CopY operator to be larger and more selective than the canonical *cop* box motif. However, our bioinformatics data indicate that there is strong conservation across the protein family operators (Fig. 8B). We hypothesized that some degree of cross-species interactions or substitution could be possible *in vitro*. Therefore, we produced recombinant CopY from both *E. hirae* and Streptococcus thermophilus to further test our proposed operator consensus and to glean details on how the proteins bind to their operators. We encountered problems with initial purification of *E. hirae* CopY. Incubation with tobacco etch virus (TEV) protease led to proteolysis. The ideal sequence for TEV protease cleavage is ENLYFQ | GS, with the pipe character representing the hydrolyzed bond. Since this site is so specific, it is commonly used for precision proteolysis, e.g., removing a purification tag. Reviewing the amino acid sequence of *E. hirae* CopY, we identified a functional TEV site (ENLFSH | IC). After reviewing the literature, we introduced an E86D mutation (DNLFSHIC) (54). This point mutation was sufficient to abrogate the undesired proteolysis while retaining the negative charge at position 86.

We used BLI to measure binding between the S. pneumoniae, S. thermophilus, and *E. hirae cop* repressor proteins and CopY operator sites from S. pneumoniae, S. thermophilus (proximal and distal), and *E. hirae* (proximal and distal). These experiments comprised a small matrix of interactions between related CopY proteins and operators. The *E. hirae cop* repressor bound to all five operators with no detectable difference even between uncleaved *E. hirae* CopY and cleaved E86D protein, implying that the His tag and TEV cleavage site intact had no effect on binding this specific system. We found that S. pneumoniae CopY did not bind to the distal *E. hirae* operator but did bind to the *E. hirae* proximal operator and both distal and proximal *cop* operators from S. thermophilus (Tables 1 and 3). The S. thermophilus *cop* operon repressor bound to its own operators and the *E. hirae* operators (albeit weakly to the distal site) but not to the pneumococcal operator (Table 3). Taken together, the consensus sequence for these operators would be RNYKACANNTGTMRNY.

**TABLE 3:** Cross-species binding of CopY *in vitro*

CopY protein	Binding^*a*^				
	*E. hirae*		S. pneumoniae	S. thermophilus	
	Distal	Proximal		Distal	Proximal
*E. hirae*	++	++	++	++	++
S. pneumoniae	−	++	++	++	++
S. thermophilus	+	++	−	++	++

a CopY from different bacteria was assessed for its ability to bind the operators found in other bacterial species to produce a binding matrix. Interactions were scaled based on relative affinities of binding. −, no binding; +, weak binding; ++ strong binding.

## DISCUSSION

Previous binding studies characterizing pneumococcal CopY were carried out with DNA containing only one operator (21, 34, 35). Here, we demonstrate that CopR-CopY homologs in *Streptococcus* and many other genera have two operators upstream of the *cop* operon (Fig. 1A). Despite having two identical 21-base repeat operators within close proximity, the S. pneumoniae CopY protein does not have a significantly higher affinity for DNA with two operators present. A limitation of this study and subject to future direction is how these two operators affect gene regulation. Based on predicted −35 and −10 sites, we suggest that the distal operator prevents sigma factors from binding at the *copY* −35 element and that the proximal operator occludes RNA polymerase, establishing two layers of repression for the *cop* operon (55). This reasoning is also consistent with the proposed hypothesis of why there are two operators in the antibiotic resistance repressor BlaI (56). It is unclear which sigma factor drives *copY* transcription or whether the binding between CopY, a sigma factor, and/or RNA polymerase is competitive. Another subject of future direction is why such repression through multiple operators is necessary for the *cop* operon especially considering that (i) *cop* operon upregulation is linked to increased pneumococcal survival in the host and (ii) a Δ*copY* mutant has increased virulence in mice (30, 57). We anticipate that further study of these systems via fitness testing and exploring the effect of having these systems on under metal-limiting conditions will yield clues as to the competitive advantages or selective pressures of the *cop* operon at the host-microbe interface.

Given the previous 10-base consensus sequence suggested for CopR-CopY protein operators, it was plausible there were additional genes that the *cop* repressor could regulate, similarly to L. lactis (44). Some of these putative binding sites in S. pneumoniae corresponded to putative promoter regions of genes and operons upregulated under copper stress, suggesting that CopY was a regulator of several operons (14). However, we have empirically demonstrated that these putative sites—despite showing copper-dependent regulation—did not bind CopY and thus, importantly, that the canonical 10-base *cop* box is not sufficient for binding (14, 21). These facts ultimately led us to explore and propose an expanded CopY operator sequence (RNYKACAAATGTMRNY), adding to the previously reported KACANNTGTA (Fig. 8). Unlike in the L. lactis genome, a search using this operator motif in multiple streptococcal species for potential operators yielded no additional sites (48).

While CopY does not bind to other locations within S. pneumoniae, its aforementioned role in controlling the *cop* operon and the importance of that operon at the host-pathogen interface makes it of continued interest. It is still not known how CopY has higher binding to DNA with zinc bound than in the apo form and how copper changes the structure to disrupt DNA binding. This expanded operator can better inform structure/function studies regarding residue-to-nucleotide interaction in the CopR-CopY family of proteins, for which there is still no structure with metal or DNA bound.

In generating the list of homologous CopR-CopY genes and proteins, we also were able to look upstream of the gene and compile what we believe to be a consensus operator site which differs slightly from the proposed pneumococcal operator. The strong conservation between repressor and operator across species is made clear by the affinity studies with *cop* repressors from both *E. hirae* and S. thermophilus. However, this cross-species interaction was not universal—pneumococcal CopY only bound to one *E. hirae* operator but bound both S. thermophilus sites—the new consensus operator is more predictive of these proteins binding in this limited homology matrix. Based on the strong interactions we observed *in vitro*, the selectivity of repressors for operators, and a suite of predicted CopR-CopY operators, we propose a consensus operator of RNYKACANNYGTMRNY (Fig. 8). This consensus operator is consistent with the results presented by Magnani et al. using the L. lactis CopR protein (Table 2) (44).

We propose that, moving forward, operator sequences for transcriptional regulators should be revisited and examined with a higher level of scrutiny, specifically those that are just apparent palindromic sequences. In doing so, we believe that this would reveal potential binding interactions within the organism’s respective genome and with genomes of the cohabitant bacteria of their environments from which they might acquire new genes.

## MATERIALS AND METHODS

### Aligning and comparing CopY homologs and promoter sequences.

The BLAST sequence alignment algorithm was used to align both *E. hirae* and TIGR4 S. pneumoniae *cop* operon promoter regions, the 21-base repeats upstream of the TIGR4 pneumococcal *cop* operon, and the promoter regions of pneumococcal species (46). A set of custom Python scripts (available from https://github.com/Van-Doorslaer/O-Brien_et_al_2019) were used to assign identified *copY* homologs to bacterial genomes and extract the suspected regulatory region from individual species (100 and 500 bases upstream of the start codon). Importantly, in many cases, the initial BLAST search identified CopY homologs which matched multiple species isolates/strains. In this case, the identified proteins were again compared to the NCBI database, and the homolog with the lowest E value was retained. If this approach was unsuccessful, the homolog was excluded from further analysis.

### Cloning and site-directed mutagenesis.

*copR-copY* from S. pneumoniae, S. thermophilus, and *E. hirae* were cloned from genomic DNA of the respective organisms and inserted into the pMCSG7 vector (58). The PrimerX tool (http://bioinformatics.org/primerx) was used to generate primers for site-directed mutagenesis of E86D in the *E. hirae copR* gene. Recombinant Pyrococcus furiosus (*Pfu*) polymerase was used to perform all molecular reactions. Correct sequences were verified by Sanger sequencing before use.

### Protein purification.

Recombinant CopR-CopY proteins were purified as described by Neubert et al. (34), with modifications. The pMCSG7 vector includes an N-terminal 6×His tag linked to CopR-CopY via a tobacco etch virus (TEV) protease cleavage site (58). Unless otherwise specified, all steps were performed on ice or at 4°C. After initial purification using immobilized metal-affinity chromatography (IMAC) (HisTrap FF; GE Healthcare), the crude CopR-CopY sample was incubated at 23°C with a 100:1 mass ratio of recombinant TEV. The cleaved product was confirmed via SDS-PAGE. Cleaved CopR-CopY was purified with subtractive IMAC; the TEV protease contains a C-terminal His tag. The flowthrough was concentrated using concentrator with a molecular weight cutoff (MWCO) of 10 kDa (MilliporeSigma) and further purified by size exclusion chromatography (SEC) (Superdex 200; GE Healthcare) using a buffer of 20 mM Tris (pH 8), 200 mM NaCl, and 1 mM Tris(2-carboxyethyl)phosphine (TCEP). Based on the elution volume and comparison to an SEC standard (Bio-Rad), we observed that CopR-CopY proteins eluted exclusively as a dimer. Peaks containing pure CopR-CopY (as determined by SDS-PAGE) were pooled, and the concentration was determined by absorbance at 280 nm. Samples were used immediately or protected with 35% to 50% glycerol and aliquoted into thin-walled PCR tubes and flash-frozen using liquid N_2_.

### Electromobility shift assay.

Primers for binding, 5′-TAATTGACAAATGTAGATTTTAAGAGTATACTGATGAGTGTAATTGACAAATGTAGATTTT-3′ and 5′-AAAATCTACATTTGTCAATTACACTCATCAGTATACTCTTAAAATCTACATTTGTCAATTA-3′, were annealed by heating a solution containing 1:1 molar equivalents of each strand to 95°C and then reducing the temperature by ∼1°C/min to 22°C. EMSA buffer was Tris-borate (TB) electrophoresis buffer (EDTA was omitted to diminish metal chelation). Samples were incubated at 4°C for 5 min, loaded onto a 5% polyacrylamide Tris-borate-EDTA (TBE) gel (Bio-Rad) that had been prerun for 15 min in TB buffer. Samples were electrophoresed at 40 V for 120 min. The polyacrylamide gel was stained with 0.02% ethidium bromide (Amresco) and imaged with a Gel Doc XR+ system (Bio-Rad).

### Biolayer interferometry DNA/protein binding.

Double-stranded DNA fragments were hybridized as described above by incubating 5′ biotinylated ssDNA with an unlabeled complementary strand at 95°C for 5 to 10 min and then left to cool to room temperature (see Table S1 in the supplemental material). The biotinylated dsDNA oligonucleotides were diluted to 250 or 50 nM in the assay buffer (50 mM Tris [pH 7.4], 150 mM NaCl, 4% glycerol, 1 mM TCEP, 0.1% bovine serum albumin [BSA]). During initial optimization, we observed significant nonspecific binding of CopY to the biosensors in the absence of BSA. The inclusion of 0.1% BSA eliminated signals of nonspecific CopY binding at and above the highest concentrations used in the assay (3 μM). The dissociation constant for CopY with various DNA fragments was determined using an Octet Red384 (Pall ForteBio). Streptavidin biosensors (Pall ForteBio) were hydrated at 26°C using the Sidekick shaker accessory for 10 min at 1,000 rpm. Hydrated sensors were incubated in the assay buffer to acquire a primary baseline. The sensors were then loaded with biotinylated dsDNA, followed by a secondary baseline measurement using wells with buffer solution. DNA-loaded biosensors were then moved to wells containing various CopY concentrations to measure the association and then placed back into assay buffer for dissociation recordings. All experiments were maintained at 26°C with shaking at 1,000 rpm. The optimized protocol was as follows: primary baseline, 60 s; DNA loading, 150 s; secondary baseline, 180 s; association, 180 s; and dissociation, 270 s. The methods were optimized to smooth the signal at association and minimize recording at equilibria. Additionally, proteins were tested both with and without cleaving the N-terminal His tag.

10.1128/mSphere.00411-20.4 TABLE S1 List of primers used for BLI. Each of the primers in the table has a 5′ biotinylation and was the forward primer used in the dsDNA for BLI assays. Complementary reverse primers for each fragment were used to generate the dsDNA (not shown). Download Table S1, PDF file, 0.1 MB. Copyright © 2020 O’Brien et al. 2020 O’Brien et al. This content is distributed under the terms of the Creative Commons Attribution 4.0 International license.

Analysis was performed using Octet software. We applied a 1:1 binding model using a global fit to biosensor replicates at each concentration of CopY. During preprocessing, an average of the secondary baseline across the various biosensors was applied, as well as Savitzky-Golay filtering to reduce noise. The data were interstep corrected using an alignment to the dissociation step. Data were modeled using combined fits of absorption rate constant (*k_a_*) and dissociation rate (*k_d_*) values across independent replicates. Final estimates for *K_d_* and related statistics were taken from the kinetic analysis.

Rate constants for each sample were determined using the Octet analysis software as follows. For all 1:1 stoichiometric modeling, complex formation was evaluated as pseudofirst-order kinetics. The observed rate constant (*k*_obs_) was calculated according to the equation $\text{Y}={\text{Y}}_{0}+\text{A}(1-{\text{e}}^{–k_{\text{obs}}\times t})$. Where Y_0_ is the initial binding, Y is the level of binding, *t* is time, and A is asymptote value at max response. Dissociation rate (*k_d_*) was calculated according to the equation $\text{Y}={\text{Y}}_{0}+{\text{Ae}}^{–k_{d}\times t}$. The calculated k*_obs_* and k*_d_* values were then used to determine k_a_ using the equation $k_{a}=\;\frac{k_{\mathrm{obs}}-k_{d}}{\left[\mathrm{CopY}\right]}$. Finally, the dissociation constant (*K_d_*) was determined by the identity $K_{d}=\;\frac{k_{d}}{k_{a}}$.

10.1128/mSphere.00411-20.3 FIG S3 Affinity of CopY for 16-bp fragment. Binding affinity of CopY to the 16-base fragment was determined using the following concentrations of protein: 1,000 nM (red), 500 nM (orange), 250 nM (yellow), 125 nM (green), 62.5 nM (blue), 31.3 nM (purple). Download FIG S3, PDF file, 0.3 MB. Copyright © 2020 O’Brien et al. 2020 O’Brien et al. This content is distributed under the terms of the Creative Commons Attribution 4.0 International license.
