# Supplementary figures and images for: Rules of Expansion: an Updated Consensus Operator Site for the CopR-CopY Family of Bacterial Copper Exporter System Repressors
Source: mSphere. 2020 May 27;5(3):e00411-20. doi: 10.1128/mSphere.00411-20 (PMC7253601; doi:10.1128/mSphere.00411-20)

37 **Supplemental Figure 1**  
38

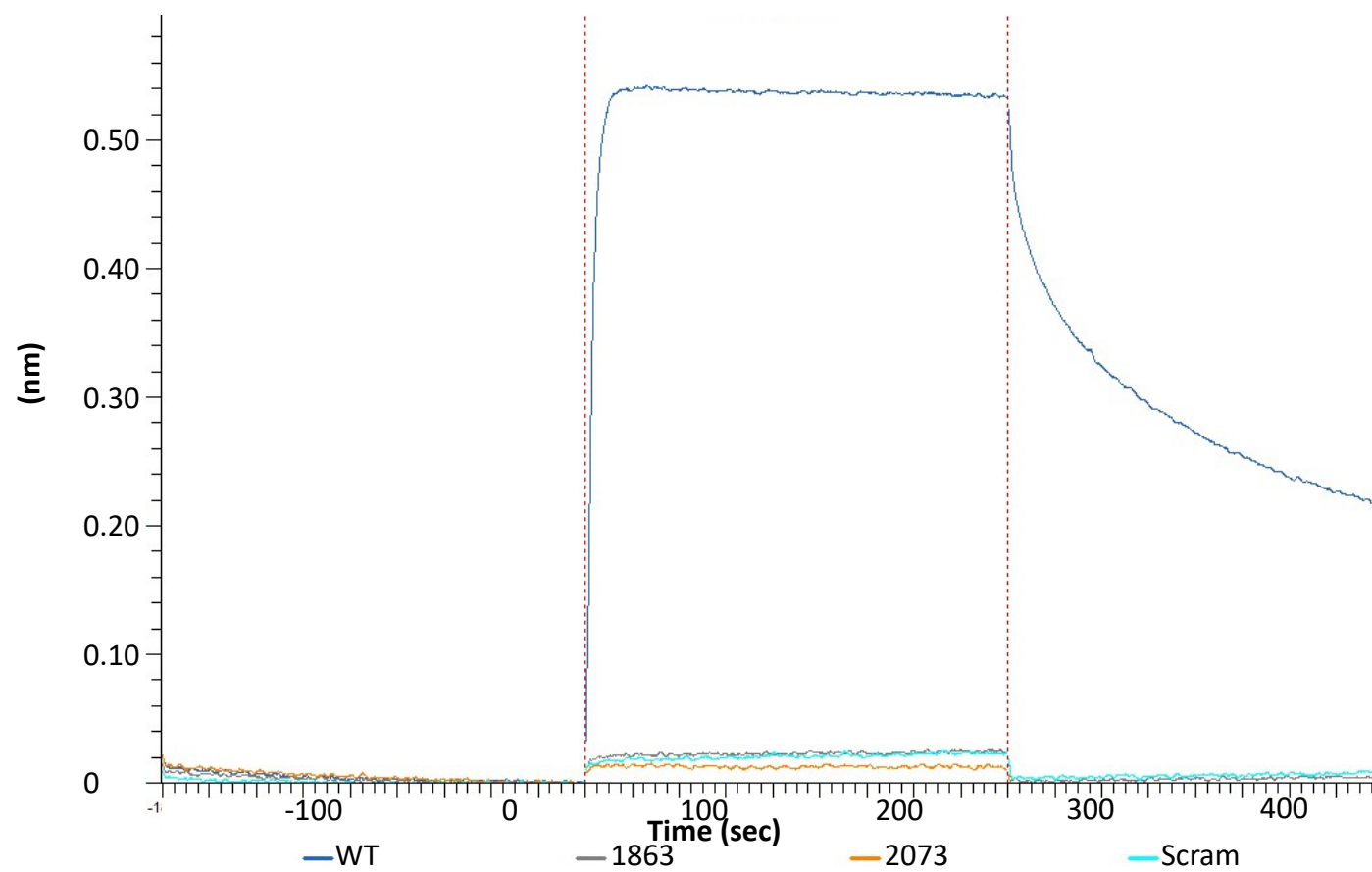

Supplement: FIG S1 [file mSphere.00411-20-sf001.pdf]

Supplemental Figure 2

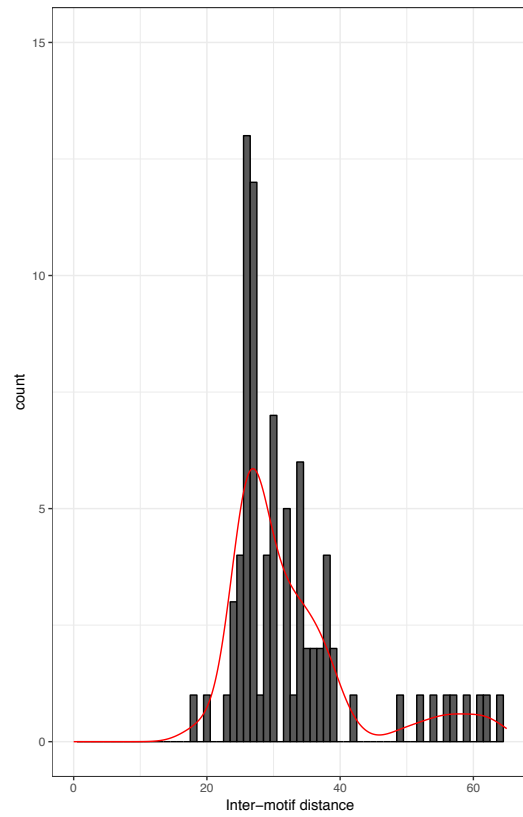

Supplement: FIG S2 [file mSphere.00411-20-sf002.pdf]

43 **Supplemental Figure 3**

44

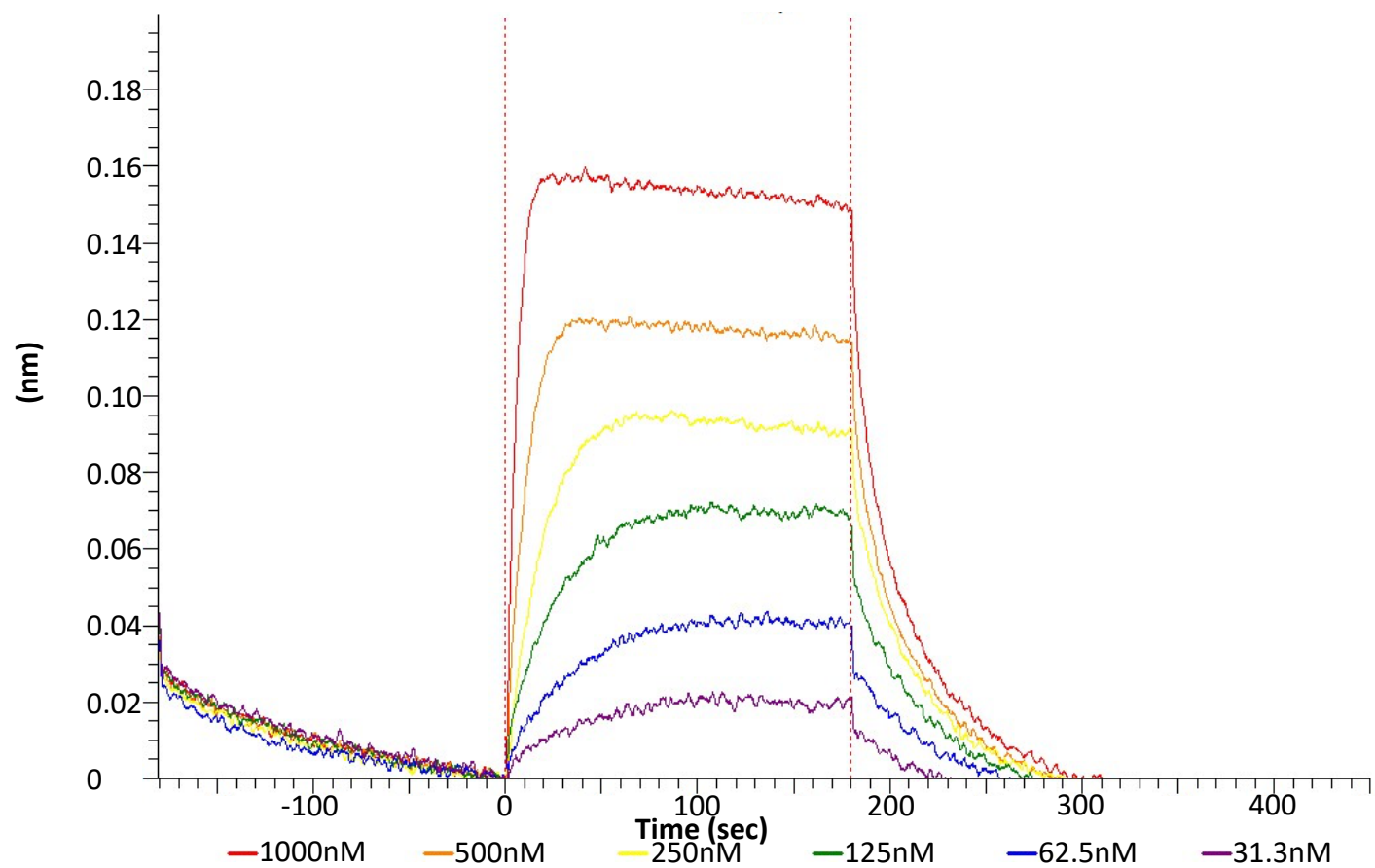

45

Supplement: FIG S3 [file mSphere.00411-20-sf003.pdf]
