# Supplementary material for: Rules of Expansion: an Updated Consensus Operator Site for the CopR-CopY Family of Bacterial Copper Exporter System Repressors
Source: mSphere. 2020 May 27;5(3):e00411-20. doi: 10.1128/mSphere.00411-20 (PMC7253601; doi:10.1128/mSphere.00411-20)
Supplement: TABLE S1 [file mSphere.00411-20-st001.pdf]

Supplemental Table 1

Primer Name	Sequence (5' biotin)
S. pneumoniae Two-Site	**TAATTGACAAATGTAGATTTT**AAGAGTATACTGATGAGTG**TAATTGACAAATGTAGATTTT**
CopY Proximal Site	ATGTATAATATAACGTTGGAATTAATATGACTGATGAGTG**TAATTGACAAATGTAGATTTT**
CopY Distal Site	**TAATTGACAAATGTAGATTTT**AAGAGTATACTGATGATTGTTATATTAGTATAGATGCAA
Scram	GTGTATTAGTTAACAAGTTATATAGTATACAAAGGATTGTGTTGATTAATAAGATTCAGTA
10-base	GTGTATTAGTTAACAAGTTATATAGTATACAAAGGATTGTGTTGA**GACAAATGTA**TCAGTA
14-base	GTGTATTAGTTAACAAGTTATATAGTATACAAAGGATTGTGTT**TTGACAAATGTAGA**AGTA
16-base	GTGTATTAGTTAACAAGTTATATAGTATACAAAGGATTGTGT**ATTGACAAATGTAGAT**GTA
19-base	GTGTATTAGTTAACAAGTTATATAGTATACAAAGGATTGTG**AATTGACAAATGTAGATTT**A
5-base Upstream	GTGTATTAGTTAACAAGTTATATAGTATACAAAGGATTGT**TAATTGACAAATGTA**TCAGTA
6-base Downstream	GTGTATTAGTTAACAAGTTATATAGTATACAAAGGATTGTGTTGA**GACAAATGTAGATTTT**
19-base T to C	GTGTATTAGTTAACAAGTTATATAGTATACAAAGGATTGTG**AATCGACAAATGTAGATTT**A
19-base T to A	GTGTATTAGTTAACAAGTTATATAGTATACAAAGGATTGTG**AATAGACAAATGTAGATTT**A
E. hirae Distal Site	GT**TTTCGATTACAGTTGTAATCTATTAT**ACAAAGGATTGTGTTGATTAATAAGATTCAGTA
E. hirae Proximal Site	GTGTATTAGTTAACAAGTTATATAGTATACAAA**TTAAGTTTACAAATGTAATCGATGGA**TA
SP_0090 1	GAGATAAAATAAACAATTGATTTAG**GACATTTGTT**TGATAGTGGTGATAAAGTTTTAAATC
SP_0090 2	GCAAATAGGAGTATAC**TAATAATGTA**ATCGTTATCAAAAGTCTAAAAAAGAATTTT
SP_0045	TCACTCTGTAGAGAACTAGGTGAACT**AACAGATGTT**TACGAAATTGTCTGGGAAAAGTTGC
SP_0530	CCACAGTCTCTCGCATCTATTTGAGG**AACAAATGTA**CGTTTATAAGAAGTCATTGGCAATT
SP_1433	TTCAAAACTATTGTATTAGTAATTAT**AACAGATGTA**TAATAGAAAAGCAATGATAGATATT
SP_1863	ATTTATTTCGATATCGAAATGAATAA**AACAATTGTA**ACACTCATCGTTCTAACTGTCAACT
SP_2073	TACAAGAGGCTCATTGACAAGGCGGA**AACATGTGTC**AATGACTTGATGGCTGAGTTCAACT
